# Familial occipital neuralgia with sporadic NIN: a reply

**DOI:** 10.1007/s10194-011-0383-8

**Published:** 2011-09-04

**Authors:** Yu Wang, Chuan-Yong Yu, Lin Huang, Franz Riederer, Dominik Ettlin

**Affiliations:** 1 Department of Neurology, Epilepsy and Headache Group, The First Hospital of Anhui Medical University, Jixi Road 218, Hefei, 230022 China; 2 Department of Neurology, University Hospital Zurich, Plattenstrasse 11, 8032 Zurich, Switzerland

Dear Sir,

We thank Dr. Alex Alfieri and Dr. Christian Strauss for their interest and comments on our article [1]. They raise the appropriate question if the familial clustering of occipital neuralgia (ON) and intermedius nerves neuralgia (NIN) might be coincidental rather than due to a common pathophysiological correlate.

We agree that the occipital nerve and the nervus intermedius have different morphological, anatomical, embryological and functional characteristics. We do not understand, however, why these differences were to exclude a common pathophysiology, namely regarding potential dysfunction of Nav1.7 sodium-channels? The few Swiss and Chinese family cases reported [1, 2] have insufficient statistical power to draw a final conclusion in either way, i.e. sporadic NIN or NIN linked to ON. We speculate that NIN, in our report, is not a sporadic familial occurrence based on the following observations: (1) NIN is an extremely uncommon neuralgia and the co-existence in two members of one family is, therefore, unlikely to be a sporadic event. It might exist subclinically in the other family members as well; (2) familial clustering of neuralgia or neuropathy of many other (morphologically diverse) peripheral nerves has been observed in a nationwide database of Sweden [3]; (3) though Alfieri and Strauss themselves in an interesting paper reported on the long history of anatomical descriptions of the nervus intermedius, the anatomy of this small nerve is not yet fully elucidated [4]. The current common definition of this nerve as a purely sensory and parasympathetic branch of the seventh cranial nerve may not cover all aspects.

Thus, the answer to the question raised by Alfieri and Strauss will likely be approached by publication of additional clinical reports on a familial co-occurrence of cranial neuralgias, as well as by more detailed basic science and genetic investigations.
